# Severe Vascular and Ductal Complications of a Pancreatic Pseudocyst: A Case of Hemorrhage, Superior Mesenteric Vein (SMV) Compression, Duct Disconnection, and Portal Vein Thrombosis

**DOI:** 10.7759/cureus.100921

**Published:** 2026-01-06

**Authors:** Hira Khan, Karim Al Annan, Khaled H Husain, Nancy Kang

**Affiliations:** 1 Internal Medicine, University of Connecticut Health, Farmington, USA; 2 Gastroenterology, Saint Francis Hospital and Medical Center, Hartford, USA

**Keywords:** acute pancreatitis, disconnected pancreatic duct syndrome, endoscopic ultrasound (eus), hemorrhagic pancreatic pseudocyst, pancreatic pseudocyst (ppc)

## Abstract

Pancreatic pseudocysts (PPCs) are a common complication of chronic pancreatitis, particularly in alcohol-related disease. While many pseudocysts resolve spontaneously, enlarging or symptomatic collections may lead to hemorrhage, vascular compression, thrombosis, or disconnected pancreatic duct syndrome (DPDS), a condition increasingly recognized in patients with necrotizing pancreatitis.

A 62-year-old man with chronic alcohol-related pancreatitis and recurrent PPCs presented with several weeks of severe epigastric pain radiating to the back. Computed tomography (CT) imaging revealed two enlarging pseudocysts (6.6 cm and 5.3 cm) in the uncinate process, with severe superior mesenteric vein (SMV) compression, fat stranding, new hemorrhage into a pseudocyst, upstream pancreatic duct dilation, and right portal vein branch thrombosis. Symptoms improved with supportive care, and outpatient endoscopic evaluation was pursued. Endoscopic ultrasound (EUS) and endoscopic retrograde cholangiopancreatography (ERCP) demonstrated walled-off necrosis and active contrast extravasation in the pancreatic neck, confirming pancreatic duct disconnection. Due to the gastroduodenal artery encircling the collection, cyst-gastrostomy was deemed unsafe. Instead, a limited pancreatic sphincterotomy was performed, followed by placement of a straight pancreatic duct stent across the disconnection. The patient had subsequent resolution of his pseudocysts and continues to be followed as an outpatient.

This case illustrates how PPCs in chronic pancreatitis can lead to multiple severe complications, emphasizing the need for early detection and prompt endoscopic management to prevent recurrent collections and reduce morbidity. It further highlights the potential progression from pseudocyst formation to DPDS, where timely recognition of ductal disruption is crucial for restoring ductal continuity, limiting recurrence, and avoiding additional complications.

## Introduction

Pancreatic pseudocyst (PPC) remains a common complication of pancreatitis, with prevalence reaching 20%-30% in patients with chronic pancreatitis [1]. Most pseudocysts are discovered incidentally on abdominal imaging, and depending on the cyst size and number, the clinical presentation is variable, ranging from patients being asymptomatic to having major complications, both acute and chronic [2]. While many pseudocysts remain asymptomatic or resolve spontaneously, others can enlarge, become infected, compress adjacent structures, or lead to recurrent pancreatitis, making timely diagnosis and management essential. In chronic pancreatitis, ongoing ductal inflammation and fibrosis increase the risk of persistent or recurrent fluid collections and may predispose patients to more complex sequelae.

Among the most challenging complications is disconnected pancreatic duct syndrome (DPDS), a condition in which a segment of viable pancreatic tissue becomes separated from the main pancreatic duct due to ductal disruption or necrosis [3]. DPDS often leads to recurrent or persistent collections, external fistulas, or non-resolving pancreatitis and is frequently underrecognized in its early stages. Because management differs from that of simple pseudocysts, failure to identify ductal disconnection can result in prolonged morbidity and repeated interventions.

This case describes a patient with chronic pancreatitis who developed multiple enlarging PPCs with progressive complications, ultimately revealing underlying DPDS. The clinical course underscores the importance of early recognition of ductal disruption, appropriate use of endoscopic ultrasound (EUS)-guided drainage, and the need for ongoing surveillance in patients with chronic pancreatitis who present with recurrent or evolving fluid collections. This case highlights diagnostic challenges and reinforces the role of timely endoscopic intervention in preventing recurrent disease and reducing long-term morbidity.

## Case presentation

A 62-year-old male with a past medical history of chronic alcohol-related pancreatitis, complicated by multiple PPCs on magnetic resonance imaging (MRI) surveillance, hypertension, obstructive sleep apnea, and osteoarthritis, presented from home with severe intermittent abdominal pain radiating to his back that had been ongoing for several weeks. This was associated with abdominal pressure and nausea. He was admitted for management of acute pancreatitis and found to have a multitude of complications related to PPCs. Vitals were notable for a heart rate of 95 bpm, blood pressure of 190/93 mmHg, and SpO₂ 98% on room air. Abdominal tenderness in the epigastric region was noted on physical examination, without any signs of peritoneal irritation. Pertinent lab findings are included in Table 1.

**Table 1:** Laboratory findings on admission with reference ranges

Lab Test	Value/Units	Reference Range
White Blood Cell Count	14.0 × 10³/μL	4.0-11.0 × 10³/μL
Blood Urea Nitrogen (BUN)	11 mg/dL	7-20 mg/dL
Amylase	1397 U/L	30-110 U/L
Lipase	7687 U/L	10-140 U/L
Alkaline Phosphatase	117 U/L	44-147 U/L

**Figure 1:**
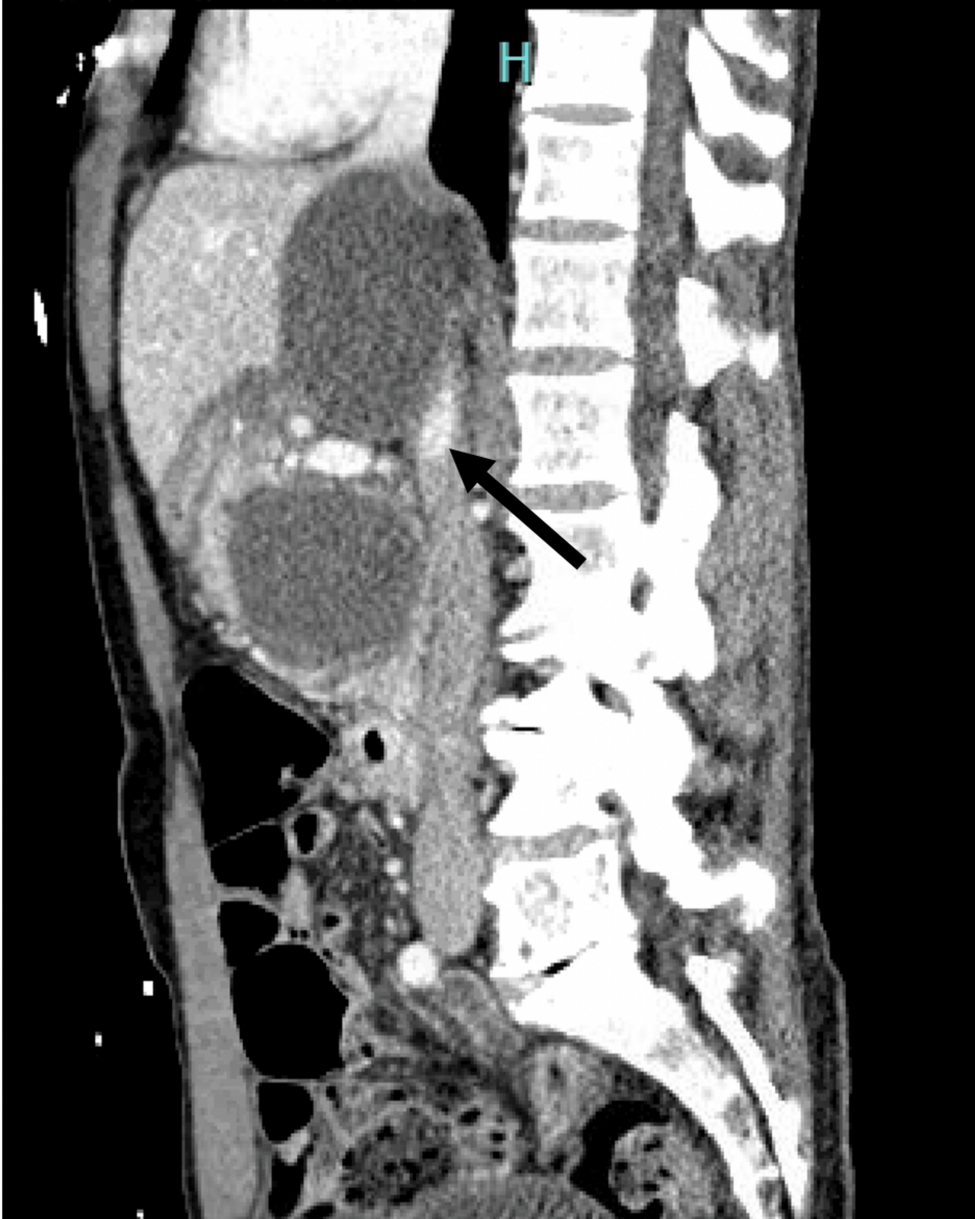
Sagittal CT abdomen and pelvis showing severe superior mesenteric vein compression (arrow) CT, computed tomography

Initial computed tomography (CT) imaging revealed a superior PPC measuring 6.6 cm and an inferior PPC measuring 5.3 cm, both centered in the uncinate process of the pancreas and extending to the pancreatic neck. The inferior PPC was significantly larger when compared to previous outpatient imaging (4.5 cm), and there was new evidence of hemorrhage into the pseudocyst, upstream dilatation of the pancreatic duct, and thrombosis of the right branch of the right portal vein (Figure 1). 

**Figure 2:**
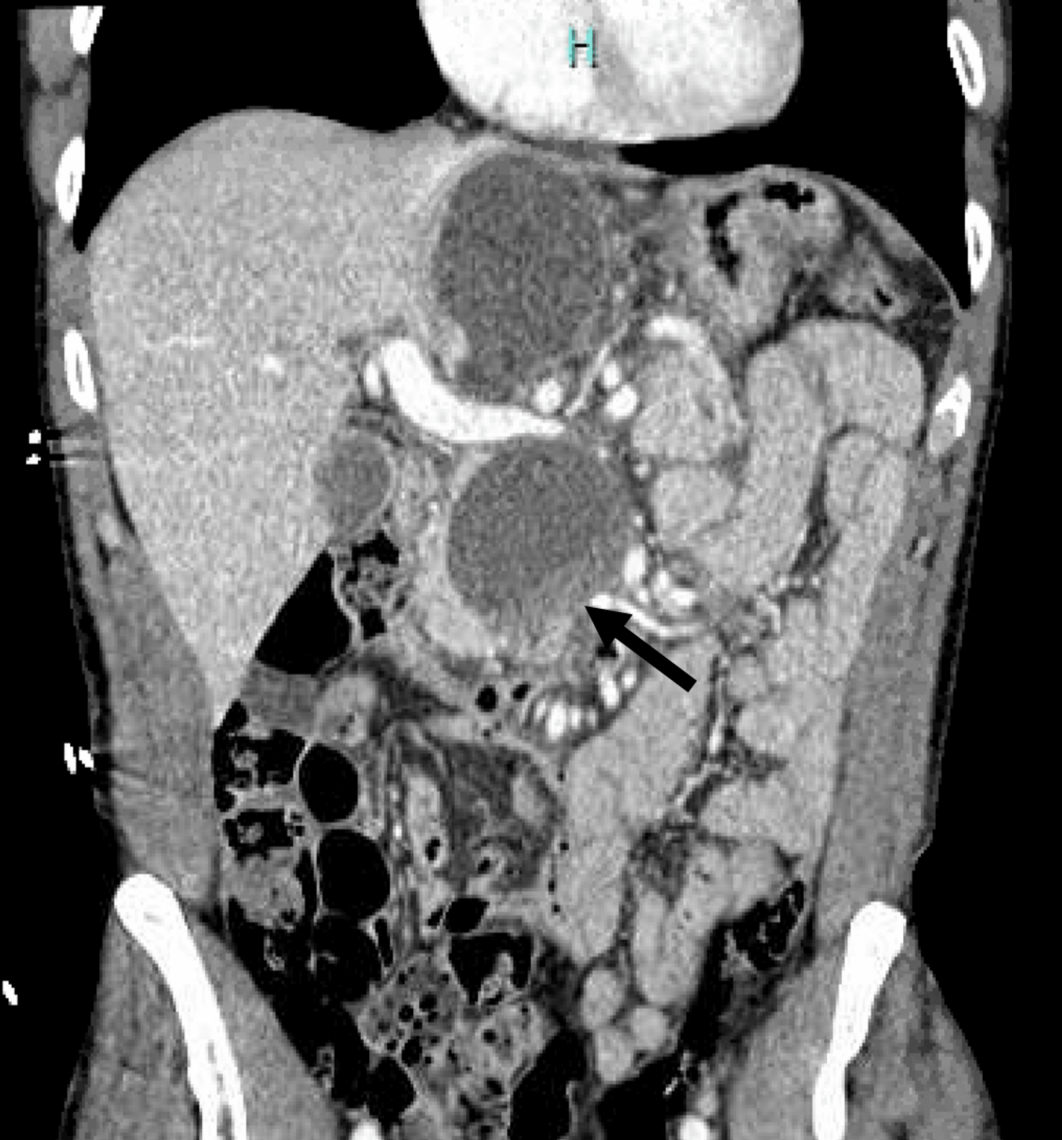
Coronal section of CT abdomen and pelvis showing a superior PPC measuring 6.6 cm, and an inferior PPC measuring 5.3 cm, with new evidence of hemorrhage (arrow) into the inferior pseudocyst CT, computed tomography; PPC, pancreatic pseudocyst

There was also severe superior mesenteric vein (SMV) compression by the superior pseudocyst, and fat stranding along the pancreas indicative of acute pancreatitis (Figure 2).

**Figure 3:**
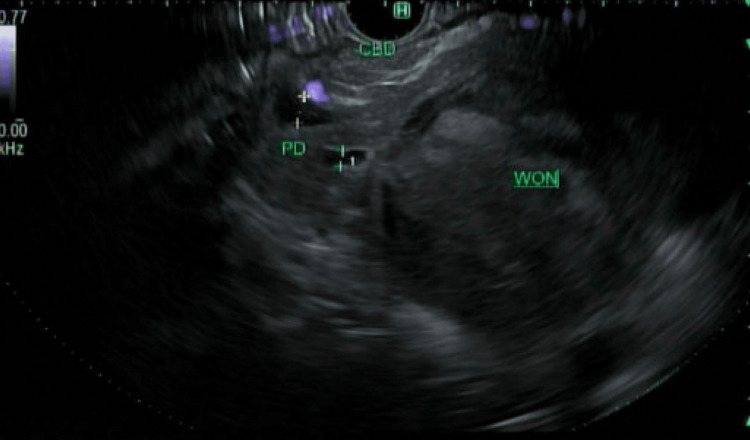
Endoscopic ultrasound image showing walled-off necrosis (WON) and active extravasation in the neck of the pancreas, suggesting pancreatic duct disconnection PD, pancreatic duct; CBD, common bile duct

The patient was treated for acute pancreatitis with intravenous hydromorphone for pain control and intravenous fluids. His symptoms improved, and he was discharged home with a plan to follow up with outpatient gastroenterology for further management. Upper endoscopy, EUS, and endoscopic retrograde cholangiopancreatography (ERCP) were performed, which revealed that the gastroduodenal artery seemed to be coursing around and wrapping the circumference of an anterior collection, with no safe window to perform cyst-gastrostomy. Pancreatogram showed new walled-off necrosis and active extravasation in the neck of the pancreas, suggesting pancreatic duct disconnection (Figure 3). 

A small pancreatic sphincterotomy was performed, and a straight pancreatic duct stent was placed across the disconnection. At outpatient follow-up, the patient’s PPCs had markedly decreased in size, and no further episodes of pancreatitis occurred over the following year.

## Discussion

PPCs are a common complication of pancreatitis, particularly in chronic alcohol-related disease [2,4]. Pancreatic inflammation and enzyme leakage lead to localized fluid collections that become encapsulated by granulation tissue rather than epithelium; when this persists beyond four weeks, it is defined as a pseudocyst [4]. PPCs may cause abdominal pain or pressure as they increase in size or number, and can lead to hemorrhage, rupture, infection, or compression of adjacent structures, including the biliary tree, duodenum, and portal venous system [2].

Asymptomatic pseudocysts with low malignant potential do not require intervention, and surveillance is reserved for patients who are surgically fit. The American College of Gastroenterology recommends treating symptomatic PPCs - those >3 cm, enlarging, or with concerning features - using endoscopic, surgical, or percutaneous drainage [4]. Recurrence rates exceed 16% after percutaneous drainage [5-7], higher than with endoscopic or surgical approaches.

Our patient initially presented with a 5.3-cm pseudocyst and underwent EUS-guided aspiration, which showed benign cytology. Five months later, imaging revealed interval enlargement to 6.6 cm, with severe SMV compression, hemorrhage into one pseudocyst, portal venous thrombosis, and acute pancreatitis requiring hospitalization - his third episode of acute-on-chronic pancreatitis in the setting of persistent ductal dilation.

Outpatient ERCP and EUS demonstrated walled-off necrosis and pancreatic duct disconnection. In DPDS, necrosis separates the viable upstream pancreas from the duodenum, and ongoing pancreatic secretions accumulate as recurrent fluid collections, explaining this patient’s non-resolving PPCs. DPDS is increasingly recognized after pancreatic necrosis, with reported frequencies of ~30%-40% [8,9]. Management depends on whether ductal disruption is partial or complete. Endoscopic transluminal drainage using lumen-apposing metal stents, with necrosectomy when needed, is now first-line and achieves clinical success rates of 80%-90% [10]. Transpapillary drainage is less effective in complete disconnection, but may benefit partial disruptions [11].

Our patient had a pancreatic duct disconnection at the neck of the pancreas. A pancreatic sphincterotomy was performed, followed by successful placement of a straight pancreatic duct stent across the disruption to reestablish continuity. This approach proved beneficial in our patient, leading to a significant reduction in pseudocyst size, resolution of abdominal pressure, and no recurrent episodes of pancreatitis at one-year follow-up. 

In the present case, transpapillary stenting was pursued as an initial, minimally invasive strategy, given the patient’s acute clinical status, the presence of a ductal leak amenable to endoscopic access, and the intent to temporize pancreatic fluid leakage while avoiding early surgical intervention. Although long-term resolution rates are lower in complete DPDS, transpapillary stenting may still provide short-term decompression and symptom control in selected cases [12]. Alternative management options, including endoscopic transmural drainage and surgical approaches such as distal pancreatectomy or internal drainage, were considered. However, transmural drainage carries risks of bleeding, perforation, and stent-related adverse events, while surgical intervention is associated with higher morbidity, particularly in the acute or inflammatory phase [13]. Given these considerations, transpapillary stenting was selected as an initial management approach, with the understanding that definitive therapy might be required if clinical or radiographic resolution was not achieved.

## Conclusions

This case underscores the complex interplay between chronic pancreatitis, PPCs, and DPDS, highlighting the importance of early recognition and tailored endoscopic intervention. Timely identification of ductal disruption and appropriate restoration of pancreatic duct continuity can significantly reduce recurrent fluid collections, prevent complications, and improve long-term outcomes. Our patient’s notable clinical improvement - including resolution of symptoms, decreased pseudocyst size, and absence of recurrent pancreatitis at one-year follow-up - illustrates the effectiveness of endoscopic management in selected patients. Continued awareness of evolving endoscopic techniques is essential to optimize care for individuals with chronic pancreatitis and its associated complications.
